# The Real Cure

**Published:** 1923-02

**Authors:** Roger W. Babson


### THE REAL CURE

BY ROGER W. BABSON

Many times we have diagnosed certain industrial troubles as spiritual rather than material. By that we meant that the motives and viewpoints of the people involved were wrong. Consequently they found themselves in difficulties. Probably at least two-thirds of the troubles of this world are spiritual. Certainly ninety-five per cent. of our social evils, and I hesitate to say how many of our political troubles, would immediately disappear if people really believed the religion they profess. The relations between capital and labor, the struggles between nations, and the conflict between individuals depend upon whether or not the parties involved have the right spiritual insight. Physicians are also agreed that a very large percentage of our bodily ailments would be avoided if we were at peace spiritually.

This is what we mean when we say that lack of religion is responsible for most of the world's troubles. From this standpoint it is interesting to see the tremendous amount of interest which Coué has created by demonstrating the effect of mental peace on bodily welfare. *Aside from the question of Divine healing it is assuredly a fact that the gospel of St. Paul, which most people have heard preached from their childhood, contains all and more than Coué or any other scientist has ever offered. The moment, however, that some one comes along with only a part of the same story dressed up in scientific language, every one hails it as a new discovery.*

The reason is that the majority of people have come to look upon their religious activity as an abstract duty rather than a source of practical power. Even the ministry itself seems to have lost sight of this most important phase of its work. If it had not, every minister in the country today would be on his toes proclaiming that faith in God will do all and more than Coué can offer. The fact is however, that practically all of the Christian ministry are willing to stand by and let Coué monopolize the most valuable part of their work. *It is true that the love of God is the only solution of our greatest troubles. When our churches really wake up and show people how to secure this great power we shall hear no more about lack of interest in religion!*

